# Development of a Novel Chimeric ND-GP cVLPs Vaccine for the Prevention of Goose-Derived Newcastle Disease and Gosling Plague

**DOI:** 10.3390/microorganisms12112266

**Published:** 2024-11-08

**Authors:** Jindou Li, Jiaxin Ding, Chunhong Guo, Xiaohong Xu, Chunhui Shan, Jing Qian, Zhuang Ding

**Affiliations:** 1 State Key Laboratory for Diagnosis and Treatment of Severe Zoonotic Infectious Diseases, College of Veterinary Medicine, Jilin University, Changchun 130062, China; ljd21@mails.jlu.edu.cn (J.L.); dingjx19@mails.jlu.edu.cn (J.D.); chunhong8124@163.com (C.G.); xuxiaohong@jlu.edu.cn (X.X.); sch15148932600@163.com (C.S.); 2 Institute of Veterinary Medicine, Jiangsu Academy of Agricultural Sciences, Nanjing 210014, China

**Keywords:** goose-derived ND, GP, ND-GP cVLPs, immunoprotective effect

## Abstract

Goose-derived Newcastle disease (ND) and gosling plague (GP) are serious threats to the goose industry. Conventional vaccines have made significant contributions to preventing GP and ND. Nevertheless, the renewal of conventional vaccines and the application of novel vaccines are urgently needed to align with eco-friendly and efficient breeding concepts and achieve the final goal of epidemic purification. Therefore, based on the Newcastle disease virus-like particles (ND VLPs) vector platform, we developed novel chimeric ND-GP bivalent cVLPs (ND-GP cVLPs) displaying the NDV HN protein and the GPV VP3 protein. In vivo, immunization experiments revealed that geese immunized with 30 µg, 50 µg, or 70 µg of the ND-GP cVLPs and commercial vaccines produced highly effective hemagglutination inhibitory antibodies against NDV and neutralizing antibodies against GPV, respectively. Furthermore, 70 µg of the ND-GP cVLPs effectively protected against virulent NDV and GPV, reducing tissue damage from viral infection and virus shedding in the oropharynx and cloaca. In conclusion, we provide eco-friendly and efficient novel ND-GP cVLPs for preventing goose-derived ND and GP. Our findings provide the basis for using ND VLPs as foreign protein carriers for the developing of multi-conjugate vaccines.

## 1. Introduction

Newcastle disease (ND) is an acute, febrile, septicemic, and highly contagious disease caused by the virulent strains of Newcastle disease virus (vNDV). It poses a serious threat and causes considerable economic losses in the poultry industry [1]. NDV primarily affects chickens, but its host range has significantly expanded to include 250 bird species [2] due to the ND pandemic and NDV genetic variation. Waterfowl are natural hosts of low-virulence NDV strains, which were once believed to have a natural resistance against virulent NDV strains. However, they have been identified as natural hosts of low-virulence NDV strains, and during the fourth pandemic of ND, outbreaks in geese due to genotype VII NDV infection occurred in China [3], indicating the transmission of NDV from terrestrial birds to waterfowl. A virulent NA-1 strain of genotype VII NDV, known for its lethality to nonimmune geese and chickens, was isolated by our team from geese in Jilin, China [4]. Consequently, outbreaks of ND in ducks have become more frequent [5,6], indicating the increasing threat of NDV to waterfowl. Over the subsequent decade, no characteristic variations in NDV circulating in geese were observed, with most belonging to the subgenotype VIId NDV strain. However, the subgenotype XIIb NDV strain Goose/CH/GD/E115/2017 (E115), isolated from geese in Guangdong Province, China, in 2017, has been proven to be effectively transmitted between and within species of chickens and geese through direct contact [7]. Hence, preventing goose-derived NDV is of immense significance for controlling NDV transmission in waterfowl and poultry. Currently, chicken ND vaccines, such as the La Sota, B1, and I2 strains, are predominantly used to prevent goose-derived ND, which does not match the genotypes of the circulating strains of goose-derived NDV.

Gosling plague (GP), also known as goose parvovirus disease, is an acute or subacute septicemic infectious disease caused by goose parvovirus (GPV) [8,9]. GPV primarily targets goslings and Muscovy ducks that are one month old, causing high morbidity and mortality rates. Currently, the GP vaccines used in clinical practice include goose breeding vaccines and gosling vaccines, such as the SYG and D strains. The breeding geese vaccine strain, which is slightly more virulent, is administered to geese one month before egg laying to enable goslings to acquire maternal antibodies, providing protection against GPV during the vulnerable period. Therefore, gosling vaccines are susceptible to maternal antibody interference, making it difficult to distinguish between antibodies against wild virus infection and vaccine-induced ones. Typically, GPV vaccine strains are cultivated in goose or Muscovy duck embryos. However, the current supply of specific pathogen-free (SPF) goose or Muscovy duck embryos is insufficient, posing potential risks of contamination and viral revirulence during the vaccine strain passage.

While conventional vaccines have significantly contributed to the prevention of ND and GP, the upgrading and replacement of these vaccines have emerged as crucial breakthroughs in vaccine development, fostering advancements in the poultry industry. To implement the concept of modern eco-friendly and safe breeding and increase the control of ND and GP, it is of immense significance to develop an eco-friendly and efficient novel vaccine that is consistent with the genotype of the current circulating strain. Virus-like particles (VLPs) are hollow nanostructures assembled from one or more of the structural proteins of viruses. VLPs have been extensively developed and used in several viral vaccines due to their favorable safety profile and immunogenicity, attributed to their structural similarity to live viruses and lack of viral nucleic acid [10]. The baculovirus expression vector system (BEVS) is a mature platform for producing foreign proteins with many advantages, such as rapid development, flexible product design, high safety, scalability, and abundant posttranslational modifications [11]. Currently, several VLPs have been prepared using BEVS that have been commercialized, such as GSK Cervarix™ for Papillomavirus and B.Ingelheim CircoFLEX^®^, MSD Animal Health Porcilis^®^ PCV, and Merck Animal Health Circumvent^®^ PCV G2 for Porcine circovirus-2. Furthermore, multiple VLP vaccines are currently in the trial phase, including the Norwalk virus-VLP from Baylor College of Medicine (Houston, TX, USA) and the H1N1 2009 influenza-VLP from Novavax (Gaithersburg, MD, USA) in clinical phase II, along with the Parvovirus B19 from Meridian Life Science (Memphis, TN, USA) in clinical phase I/II.

Newcastle disease virus-like particles (ND VLPs) consist of nanoparticles formed by the assembly of NDV M, F, and HN proteins [12]. ND VLPs not only provide effective protection against the virulent strains of NDV [13] but also have been used as delivery vectors for heterologous proteins in multivaccine construction. ND VLPs mainly carry foreign proteins in two ways. The first method is the extracellular domain replacement strategy, which is the most commonly used strategy. This involves replacing the type I glycoprotein with the extracellular domain of the F protein or replacing the type II glycoprotein with the extracellular domain of the HN protein. Using this strategy, the RSV F or G protein [14,15], H9N2 AIV HA protein [16], EBV gp350/220 protein [17], and IBDV VP2 protein [1] are chimeric to the envelope the surface of ND VLPs to develop chimeric VLPs (cVLPs). Another approach involves the use of the glycosylphosphatidylinositol (GPI)-anchoring strategy. By utilizing the ability of GPI to bind to lipid rafts in the phospholipid bilayer, the *Brucella* BCSP31 protein is fused with GPI (GPI-BCSP31) and then anchored to the ND VLPs [18]. Either way, in vivo experiments showed that these cVLPs induced high levels of antibodies and offered effective protection against virus challenges. The greatest advantages of the extracellular domain replacement strategy are its simplicity and its suitability for commercial applications. The advantage of GPI is its ability to achieve the precise quantification of GPI-fused proteins anchored to the surface of VLPs, making it more suitable for anchoring molecular adjuvants to VLPs or cVLPs constructed using extracellular domain replacement strategies. For example, compared to AIV VLPs alone, GPI-GM-CSF/GPI-IL-12- or GPI-GM-CSF/GPI-IL-12-conjugated AIV VLPs significantly enhance protective immunity against both homologous and heterologous influenza virus challenges [19,20]. The VP3 protein, the predominant structural protein in GPV, can elicit the production of neutralizing antibodies [21,22], positioning it as the preferred target for GP vaccine development and diagnostic methods. In the present study, ND VLPs assembled from the M and HN proteins of the goose-derived NDV NA-1 strain were used as vectors to deliver the GP VP3 protein and ND HN protein to construct eco-friendly and efficient chimeric ND-GP bivalent cVLPs (ND-GP cVLPs). We then assessed their immunogenicity and immunoprotective efficacy. To conclude, we present an eco-friendly and efficient novel ND-GP cVLPs vaccine candidate that confers protection against NDV and GP while also providing additional insights into a vaccine delivery platform for ND VLPs.

## 2. Materials and Methods

### 2.1. Virus, Plasmids, Cells, and Animals

The recombinant baculovirus expressing the M and HN proteins of the goose-derived NDV NA-1 strain was previously developed and stored in our laboratory [13]. Similarly, the goose-derived NDV NA-1 strain was previously isolated and stored in our laboratory [4]. The virulent GPV strain was also maintained in our laboratory. The ND-attenuated vaccine (La Sota strain) was purchased from Harbin Pharmaceutical Group Biological Vaccine Co., Ltd. (Harbin, China). The GP-attenuated vaccine (SYG41-50 strain) was obtained from Sinopharm Yangzhou Weike Biological Engineering Co., Ltd. (Yangzhou, China). The Bac-to-Bac insect–baculovirus expression system (BES) (Life Technologies Corporation, Gaithersburg, MD, USA) was maintained in our laboratory. Nonimmune 12-day-old goose embryos were purchased from a goose farm in Dehui City, Jilin Province, China. SPF 9-day-old chicken embryos were obtained from the Experimental Animal Center of Harbin Veterinary Research Institute, Chinese Academy of Agricultural Sciences. In total, 150 nonimmunized 7-day-old goslings without maternal antibodies were obtained from a goose farm in Dehui City, Jilin Province, China. During the experiment, all the goslings were housed in the Laboratory Animal Center of Jilin University. The feeding conditions included a 12 h light-dark cycle, as well as the regulation of an appropriate temperature and humidity levels, and the provision of ad libitum access to water and food. If the goslings were injured due to trampling or fighting, they would be disinfected and isolated for feeding until they recovered.

### 2.2. Construction of the Recombinant Baculovirus rBV-rVP3

Glycine–serine (GS) linkers were inserted between the intracellular and transmembrane domain genes of NDV HN and the VP3 gene to maintain protein conformation and function. The Kozak sequence (GCCACC) was inserted before the start codon to promote protein translation. Fragments of the HN intracellular and transmembrane domains (named cHN) containing part of the G_4_S sequence were amplified using the following primers: cHN-F, 5′-GTCGACGCCACCATGGACCGCGCGGTTAACAGAG-3′ and cHN-R, 5′-TCCACCACCACCAGATCCACCACCACCGTATGCCAGGGCAGCTGC-3′. The chimeric VP3 fragment (designated cVP3), containing the remaining G_4_S sequence and a 15 bp overlap with cHN, was amplified using the following primers: cVP3-F, 5′-TCTGGTGGTGGTGGATCAGGGGGTGGCGGAAGTATGGCAGAGGGAGGAGG-3′and cVP3-R, 5′-AAGCTTTTACAGATTTTGAGTTAGATATCTGGTTCCAATCAATCTATCTTCTATG-3′. To construct a recombinant transfer plasmid, named rpFastbac1-rVP3, the combined cVP3 and cHN fragments (named rVP3) were inserted into the pFastbac1 line vector digested with *Sal* I and *Hind* III. The rpFastbac1-rVP3 plasmid was then transformed into competent Escherichia coli DH10Bac cells to obtain the recombinant bacmid rBmid-rVP3. The resulting colonies were screened using blue–white spotting and confirmed by PCR. Finally, rBmid-rVP3 was transfected into sf9 cells to rescue the recombinant baculovirus (named rBV-rVP3) based on the instructions of the X-treme GENE HP DNA Transfection Reagent (Roche, Basel, Switzerland). Western blotting and indirect immunofluorescence assays were performed to validate the effective rescue of the rBV-rVP3 and normalize the expression of the rVP3 protein following a previously described method [1]. The GPV VP3 mouse polyclonal antibody used in these assays was generated and maintained in our laboratory.

### 2.3. Preparation and Identification of ND-GP cVLPs

The virus titers of rBV-M, rBV-HN, and rBV-rVP3 were assessed using the BacPAK™ Baculovirus Rapid Titer Kit (Clontech, Mountain View, CA, USA). Sf9 cells (2 × 10^6^ cells/mL) were infected with rBV-M, rBV-HN, and rBV-rVP3 at an MOI of 2:1:1 for 72 h. The ND-GP cVLPs were then collected and purified following a previously described method [13]. Subsequently, the morphological structure of the ND-GP cVLPs was observed using a Hitachi H-7650 transmission electron microscope (TEM) (Hitachi Ltd., Tokyo, Japan). Additionally, immunoelectron microscopy (IEM) was employed to analyze the HN protein and VP3 protein on the surface of the ND-GP cVLPs, following previous methods [18]. The rabbit anti-NDV HN polyclonal antibody (prepared and stored in our laboratory) and rabbit anti-GPV VP3 polyclonal antibody (prepared and stored in our laboratory) were diluted at a 1:100 ratio for this purpose. Finally, the M, HN, and rVP3 proteins comprising the ND-GP cVLPs were detected via Western blotting. The antibodies used, including the rabbit anti-NDV M polyclonal antibody, rabbit anti-NDV HN polyclonal antibody, and rabbit anti-GPV VP3 polyclonal antibody (prepared and stored in our laboratory), were diluted at a ratio of 1:2000 for the experiment.

### 2.4. Immunization and Challenge of Goslings

In total, 150 14-day-old goslings with nonimmune and nonmaternal antibodies were randomly divided into the following six groups: the ND-GP cVLPs group (30 µg, *n* = 30), the ND-GP cVLPs group (50 µg, *n* = 30), the ND-GP cVLPs group (70 µg, *n* = 30), the GP-attenuated commercial vaccine group (SYG41-50 strain, *n* = 15), the ND-attenuated commercial vaccine group (La Sota strain, *n* = 15), and the phosphate-buffered saline (PBS) group (*n* = 30). Therefore, each group of geese was considered an experimental unit. Two researchers (Jindou Li and Chunhui San) were aware of the grouping of the goslings due to their involvement in the different vaccine immunizations, while the remaining researchers were blinded. For the immunization with the ND-GP cVLPs, 100 µL of 30 µg, 50 µg, or 70 µg of ND-GP cVLPs were emulsified with Freund’s adjuvant in equal volumes and then administered to goslings via intramuscular injection. Freund’s complete adjuvant was used for the first immunization, while Freund’s incomplete adjuvant was used for the booster immunization. The commercial ND and GP vaccines were administered subcutaneously in the neck at doses of 10^6^EID_50_/0.1 mL and 10^5^ELD_50_/0.1 mL per feather, respectively, in accordance with the manufacturer’s guidelines. The immunization schedule included a primary immunization at 14 days of age and a booster immunization at 28 days of age. During this period, any accidental animal deaths, including but not limited to trampling, will be excluded. Two weeks post-booster immunization, goslings from the 70 µg of ND-GP cVLPs (*n* = 15) and the ND-attenuated commercial vaccine group (*n* = 15) were challenged with virulent strains of goose-derived NDV and GPV at doses of 10^6^ ELD_50_/0.2 mL. Goslings from the 70 µg of ND-GP cVLPs (*n* = 15) and the GP-attenuated commercial vaccine group (*n* = 15) were challenged with virulent strains of goose-derived NDV and GPV at doses of 10^6^ ELD_50_/0.2 mL. The goslings would be excluded if the virus infection resulted in their death.

### 2.5. Detection of NDV Hemagglutination Inhibition (HI) Antibodies and GPV-Neutralizing Antibodies

Weekly serum samples were randomly collected from the subwing vein of the goslings in each group (*n* = 10) following their immunization. The levels of NDV HI antibodies were evaluated as previously described [23]. GPV-neutralizing antibodies were detected through a virus neutralization test. Briefly, serum samples were first inactivated at 56 °C for 30 min. Then, 50 µL of 8-fold diluted serum was added to a 96-well plate containing 50 µL of DMEM for multiple dilutions. Then, 50 µL of GPV (100 TCID_50_/0.1 mL) was added and incubated at 37 °C for 60 min. After the aseptic collection of 12-day-old goose embryo fibroblasts (GEF), 100 µL of 1 × 10^4^ GEF cells resuspended in DMEM containing 4% fetal bovine serum was added to the virus–antibody incubation wells. The number of cytopathic wells was then counted every 24 h, and the titer of virus-neutralizing antibodies was calculated after 144 h.

### 2.6. Detection of the Protective Effect Against Challenge

Following the challenge, the survival and clinical manifestations of the goslings (*n* = 10) were monitored daily before the morning feeding, while their body weights were measured every two days before the morning feeding. Additionally, oropharyngeal and cloacal swabs from the goslings (*n* = 10) were obtained on days 3, 5, 7, 9, and 11 post-challenge (dpc) to determine viral shedding after the weighing step. Briefly, the oropharyngeal and cloacal swabs were incubated for 1 h in PBS supplemented with 1000 U of penicillin and 1000 U of streptomycin. After centrifugation at 2000× *g* for 10 min, the supernatant was transferred to a new centrifuge tube and incubated overnight at 4 °C with 1 mL of PBS containing penicillin and streptomycin. Subsequently, 0.2 mL of virus diluent from the oropharyngeal and cloacal swabs of the GPV challenge groups were inoculated into 12-day-old nonimmune goose embryos for 120 h. Allantoic fluid was obtained, genomic DNA was extracted for PCR identification, and the number of positive bands was counted. The GPV-specific primers used were as follows: VP3-F, 5′-GTGGGTAATGCCTCGGGAAA-3′ and VP3-R, 5′-ATACACATCCGACGGGAACG-3′. For the NDV challenge groups, 0.2 mL of virus diluent from the oropharyngeal and cloacal swabs were inoculated into 9-day-old SPF chicken embryos for 72 h. Allantoic fluid was collected to perform a hemagglutination (HA) test to screen for positive samples. Subsequently, genomic RNA with low or no HA titer was obtained. Following reverse transcription, cDNA was obtained for PCR identification, and the number of positive bands was quantified. The national standard primers for the NDV detection were as follows: F-F, 5′-ATGGGCYCCAGAYCTTCTAC-3′ and F-R, 5′-CTGCCACTGCTAGTTGTGATAATCC-3′.

Three goslings were randomly selected from each challenge group and humanely euthanized at 5 dpc. Lung and small intestine (duodenum) samples were obtained from the goslings in the NDV challenge groups, whereas brain (cerebrum) and small intestine (duodenum) samples were collected from those in the GPV challenge groups for pathological sectioning to observe in vivo histopathological alterations. In brief, the tissue blocks were immersed in 8% paraformaldehyde (Solarbio Science & Technology Co., Ltd., Beijing, China), dehydrated in different concentrations of alcohol, and embedded in paraffin before being sectioned. After hematoxylin and eosin (H&E) staining (Solarbio Science & Technology Co., Ltd., Beijing, China), the pathological section samples were observed and photographed using a microscope (Olympus, Tokyo, Japan).

### 2.7. Data Analysis

Graph Prism was used for image production and data analysis in this research. The Shapiro–Wilk test was used to assess the normal distribution of the data, and Student’s *t* test was used to assess the group differences. A *p*-value of <0.05 was considered to indicate statistical significance (* *p* < 0.05, ** *p* < 0.01, *** *p* < 0.001).

### 2.8. Ethical Statement

All the geese were humanely raised and treated. The animal experiments were carried out following the operating procedures approved by the Animal Welfare and Research Ethics Committee of Jilin University (Approval Code: KT202302225).

## 3. Results

### 3.1. Successfully Generated rBV-rVP3 and Correctly Expressed the rVP3 Protein

**Figure 1:**
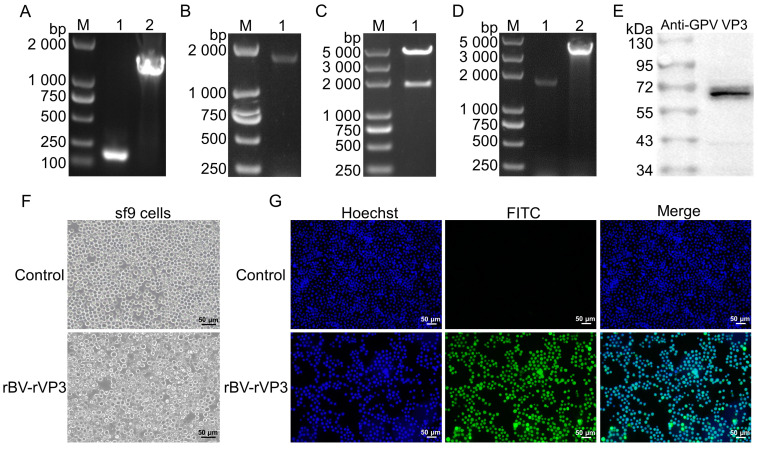
The rescue and identification of the rBV-rVP3. (**A**) The amplification of the cHN and cVP3 fragments. Lane 1 contained a 177 bp cHN fragment, while lane 2 contained a 1644 bp cVP3 fragment. (**B**) The rVP3 fragment was obtained by overlap extension PCR with the cHN and cVP3 as templates. Lane 1 contained a 1806 bp fragment of the rVP3. (**C**) A restriction analysis of the rpFastbac1-rVP3 was performed using the restriction enzymes *Sal* I and *Hind* III. The short fragment was approximately 1800 bp long for the rVP3 gene, while the long fragment was 4708 bp long for the pFastbac1 linear vector. (**D**) The rBV-rVP3 genome was identified using rVP3-specific and M13 universal primers. Lane 1 was an approximately 1800 bp rVP3 fragment, and lane 2 was an approximately 4100 bp fragment containing the Tn7 transposon gene and the rVP3 gene. (**E**) The rVP3 protein was detected via Western blotting. (**F**) The morphological examination of the sf9 cells infected with rBV-rVP3 compared to the uninfected control sf9 cells. (**G**) The immunofluorescence detection of the rBV-rVP3-infected sf9 cells compared to the control sf9 cells.

The cHN fragment (177 bp) and cVP3 fragment (1644 bp) were amplified (Figure 1A), and the rVP3 fragment (1806 bp) was obtained through overlap extension PCR (Figure 1B). Then, the rVP3 fragment was ligated to the pFastbac1 vector to construct the recombinant transfer plasmid rpFastbac1-rVP3. The resulting restriction vector was 4709 bp long, and the length of the rVP3 was 1800 bp after a restriction enzyme digestion with *Sal* I and *Hind* III (Figure 1C). The recombinant bacmid rBmid-rVP3 was obtained and transfected into sf9 cells to produce the recombinant baculovirus rBV-rVP3. Upon infection with rBV-rVP3, the sf9 cells exhibited shedding and disintegration (Figure 1F). Additionally, the rBV-rVP3 genome was extracted and confirmed by PCR. Specifically, the rVP3-specific primer amplified a fragment of approximately 1800 bp, while the M13 universal primer amplified a fragment of approximately 4100 bp (Figure 1D), which is consistent with the expected values. Then, the expression of the rVP3 protein was further confirmed. Western blotting revealed that the rVP3 protein was approximately 66 kDa (Figure 1E). Moreover, the immunofluorescence assay (IFA) results showed that, compared to the control sf9 cells, the rBV-rVP3-infected sf9 cells exhibited strong green fluorescence (Figure 1G).

### 3.2. Assembly and Identification of ND-GP cVLPs

**Figure 2:**
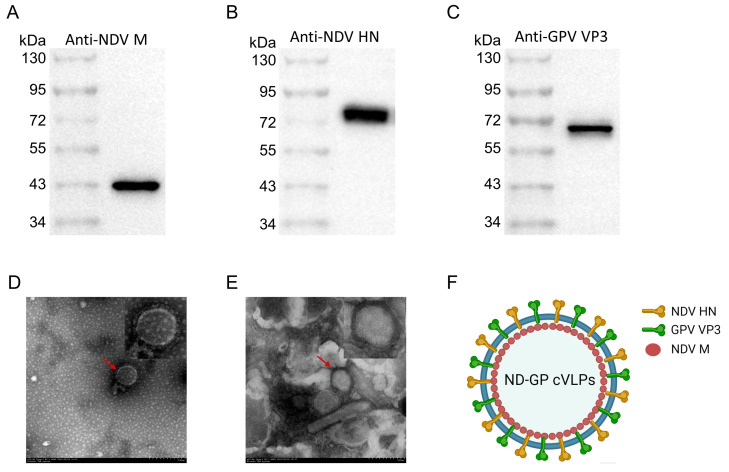
Assembly and identification of ND-GP cVLPs. (**A**–**C**) Identification of M, HN, and cVP3 proteins comprising ND-GP cVLPs through Western blotting. Analysis of morphological composition of NDV virions (**D**) and ND-GP cVLPs (**E**) by TEM. (**F**) Schematic representation of structure of ND-GP cVLPs.

Following their construction and purification, the ND-GP cVLPs were subjected to identification via Western blotting and transmission electron microscopy (TEM). The Western blotting results showed the correct expression of all the components of the ND-GP cVLPs. The M protein had a molecular weight of approximately 40 kDa (Figure 2A), the HN protein had a molecular weight of approximately 70 kDa (Figure 2B), and the cVP3 protein had a molecular weight of approximately 65 kDa (Figure 2C). Furthermore, the TEM analysis revealed that the ND-GP cVLPs possessed a nanostructure measuring approximately 100 nm with a capsule and fibril (Figure 2E), which was similar to the structure of NDV (Figure 2D). Additionally, immunoelectron microscopy (IEM) revealed the presence of 10 nm gold-labeled secondary antibodies on the ND-GP cVLPs, confirming that the HN protein and rVP3 protein can be incorporated into the membrane surface of the ND-GP cVLPs (Supplementary Figure S1). A diagram of the pattern of the ND-GP cVLPs is shown in Figure 2F, with the NDV M protein forming a polymeric core as the internal scaffold, which is enveloped by a membrane, and external fibers composed of the NDV HN and GPV VP3 proteins.

### 3.3. ND-GP cVLPs Induced Potent Humoral Immune Responses

**Figure 3:**
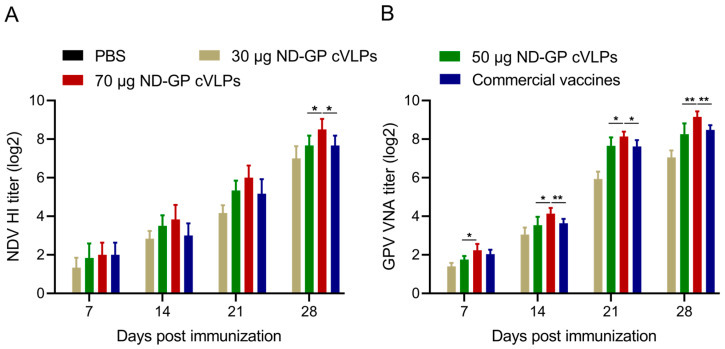
Monitoring of antibody titers induced by different immunization doses of ND-GP cVLPs. Detection of HI antibody titers against NDV (**A**) and VNA titers against GPV (**B**). A *p*-value of < 0.05 was considered to indicate statistical significance (* *p* < 0.05, ** *p* < 0.01).

The presence of HI antibodies against NDV and viral neutralizing antibodies (VNA) against GPV was evaluated within two weeks after the primary immunization and two weeks post-booster immunization, respectively. The HI test revealed that the titer of NDV HI antibodies increased with the duration and number of the immunizations. Following the primary immunization, there was minimal variation in HI antibody levels among the different immunization groups, ranging from 1.00 log2 to 3.00 log2 in the first week after the primary immunization and from 3.00 log2 to 5.00 log2 in the second week after the primary immunization. Notably, during the first week post-booster immunization, the HI antibody titers of the 70 µg of ND-GP cVLPs immunization group were the highest, averaging 6.00 log2. The HI antibody titers of the 50 µg of ND-GP cVLPs immunization group (with an average of 5.33 log2) were equivalent to those of the commercial vaccine group (with an average of 5.17 log2). Among the immunization groups, the HI antibody titer was the lowest in the 30 µg of ND-GP cVLPs group (with an average of 4.17 log2). Specifically, the average HI antibody titer induced by 70 µg of ND-GP cVLPs in the second week post-booster immunization was 8.50 log2. In comparison, the average antibody titer induced by 50 µg of the ND-GP cVLPs and the commercial vaccine group was 7.67 log2, while that induced by 30 µg of the ND-GP cVLPs was 7.00 log2 (Figure 3A). Notably, the HI antibody titers of the various doses in the VLP immunization groups and the attenuated vaccine group were >4 log2, indicating effective protection against virulent NDV challenge.

Additionally, the results of the GPV neutralizing antibody assay revealed that the average VNA titers induced by 70 µg of the ND-GP cVLPs reached 2.23 and 4.13, those induced by 50 µg of the ND-GP cVLPs reached 1.75 and 3.53, those induced by 30 µg of the ND-GP cVLPs reached 1.40 and 3.05, and those induced by the commercial GP vaccine reached 2.03 and 3.63 in the first and second weeks after the primary immunization, respectively. The VNA titers were the highest following the immunization with 70 µg of the ND-GP cVLPs, reaching 8.13 log2 and 9.15 log2 in the first and second weeks post-booster immunization, respectively. There was no significant difference in the VNA titer between 50 µg of the ND-GP cVLPs (averaging 7.65 log2 and 8.25 log2) and the commercial GP vaccine (averaging 7.62 log2 and 8.48 log2) in the first and second weeks post-booster immunization, respectively. The VNA titers induced by 30 µg of the ND-GP cVLPs were the lowest, averaging 5.9 log2 and 7.05 log2, respectively (Figure 3B). Overall, the HI and VNA titers induced by the ND-GP cVLPs were positively correlated with the immunization dose. Notably, the antibody titers induced by 70 µg of the ND-GP cVLPs were the highest among the different immunization dose groups, slightly surpassing those produced by the commercial vaccines.

### 3.4. ND-GP cVLPs Protected Geese from Death and Maintained Stable Weight Gain Against NDV and GPV Challenge

**Figure 4:**
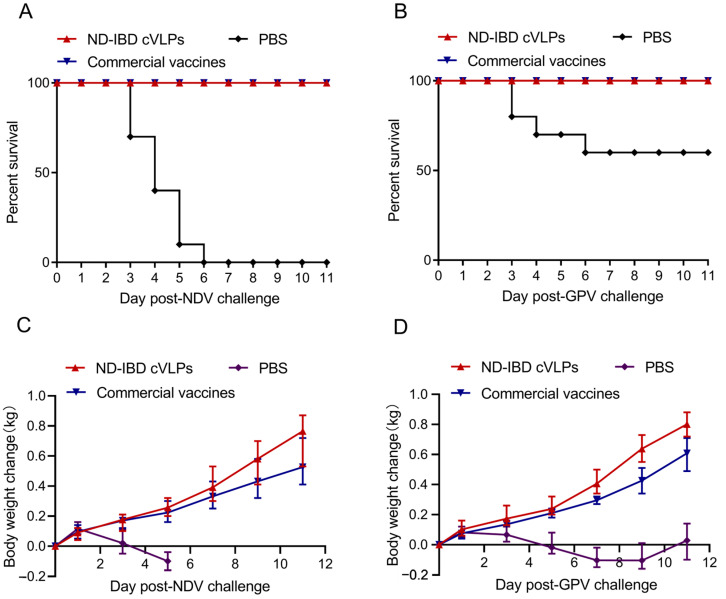
Survival rate and weight increase rate of geese immunized with 70 µg of ND-GP cVLPs after challenge. Survival rate of geese post-virulent NDV (**A**) and post-virulent GPV challenge (**B**). Weight growth rate of geese after virulent NDV (**C**) or virulent GPV challenge (**D**) was monitored.

To evaluate the immunoprotective effect of ND-GP cVLPs, the 70 µg ND-GP cVLPs immunization group, the commercial vaccine group, and the PBS group were challenged with virulent strains of NDV and GPV. The results showed that all the geese in the PBS group succumbed to the NDV challenge within 6 days, whereas all the geese in the ND-GP cVLPs and ND commercial vaccine groups were fully protected against the NDV challenge (Figure 4A). Similarly, the survival rate of the geese in the PBS group after the GPV challenge was approximately 60%, whereas all the geese in the GPV commercial vaccine and ND-GP cVLPs groups survived after the GPV challenge (Figure 4B). Furthermore, the geese immunized with the ND-GP cVLPs exhibited a faster weight gain than those immunized with the commercial vaccines following both the virulent NDV and GPV challenge (Figure 4C,D). Following the challenge with the virulent GPV, the surviving geese in the PBS group exhibited a rapid weight loss initially, followed by a gradual increase starting at 9 dpc, indicating an overall poor recovery and growth retardation (Figure 4D).

### 3.5. ND-GP cVLPs Effectively Reduced Viral Shedding in the Oropharynx and Cloaca After NDV or GPV Challenge

Oropharyngeal and cloacal swabs were collected at 3, 5, 7, 9, and 11 dpc to monitor viral shedding. At 3 dpc, NDV was detected in 40% (4/10) of the oropharyngeal swabs and 60% (6/10) of the cloacal swabs in the ND-GP cVLPs group, and in 40% (4/10) of the oropharyngeal swabs and 50% (5/10) of the cloacal swabs in the commercial vaccine group. However, NDV was detected in all the oropharyngeal swabs (10/10) and in 90% (9/10) of the cloacal swabs in the PBS group. As the dpc progressed, the number of positive samples gradually decreased until viral shedding stopped in the ND-GP cVLPs group at 9 dpc and in the commercial vaccine group at 11 dpc (Table 1). For the GPV challenge, the ND-GP cVLPs group exhibited 50% positive oropharyngeal swabs (5/10) and 60% positive cloacal swabs (6/10), whereas the commercial vaccine group showed 60% positive oropharyngeal swabs (6/10) and 70% positive cloacal swabs (7/10) at 3 dpc. The number of positive samples in the ND-GP cVLPs group and commercial vaccine group gradually decreased to zero at 5, 7, and 11 dpc in both the ND-GP cVLPs and commercial vaccine groups. Notably, the number of positive samples in the PBS group was greater than that in the commercial vaccine and ND-GP cVLPs groups, even up to 11 dpc, with a positive rate of 50% (5/10) for the oropharyngeal swabs and 60% (6/10) for the cloacal swabs (Table 2). Hence, both the ND-GP cVLPs and commercial vaccines effectively reduced viral shedding in the oropharynx and cloaca following the NDV or GPV challenge.

### 3.6. ND-GP cVLPs Provided Protection Against Target Damage Resulting from GPV or NDV Infection

**Figure 5:**
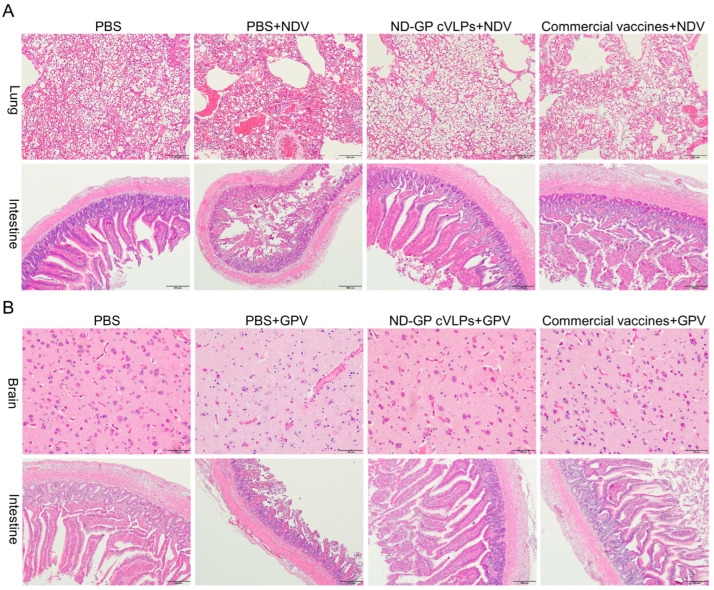
Histopathological observations of the lung and intestine after the NDV challenge and the brain and intestine after GPV challenge. (**A**) Histological observation of the lung and intestine in the PBS group, ND-GP cVLPs group, and commercial vaccine group post-NDV challenge. (**B**) Histological observation of the brain and intestine in the PBS group, ND-GP cVLPs group, and commercial vaccine group post-NDV challenge.

Because NDV infection primarily affects the respiratory and digestive tracts, we observed pathological changes in the lung and intestine on the 5th day post-NDV challenge (Figure 5A). The results revealed alveolar damage, pulmonary interstitial congestion, and inflammatory cell infiltration in the lung of the PBS group following the NDV challenge. Additionally, the shedding of the villi epithelium and a decrease in the number of intestinal villi were observed in the intestine of the PBS group. Conversely, the lung tissue and intestine of the commercial vaccine and ND-GP cVLPs groups remained structurally intact after the NDV challenge. Because GPV infection typically leads to neurological symptoms and digestive disorders, we examined pathological changes in the brain and intestine following the GPV challenge (Figure 5B). The results showed neuronal degeneration and the disappearance of Nissl bodies in the brain the exfoliation of the villi epithelium and a reduction in the villi in the intestine of the PBS group after the GPV challenge. However, the brain and intestinal structures of the commercial vaccine and ND-GP cVLPs groups remained intact after the GPV challenge, similar to those of the unchallenged PBS group. Taken together, these findings suggest that immunization with ND-GP cVLPs provides protection against tissue and organ damage caused by GPV or NDV infection.

## 4. Discussion

Goose farming is one of the most rapidly growing and economically profitable farming programs in China. It has become an integral component in some areas of animal husbandry. However, the prevalence of GP and goose-derived NDV considerably threatens the commercial goose breeding base. Attenuated or inactivated chicken-derived NDV vaccines are currently used to prevent goose-derived NDV. Similarly, the control of GP primarily depends on attenuated vaccines. To date, no approved bivalent vaccines targeting ND and GP have been developed. The limitations of traditional vaccines, including the inability to differentiate between infected and vaccinated animals and conflicts between immunogenicity and safety, highlight the urgent need for the development and application of novel vaccines that are both eco-friendly and highly efficient.

Previously, we successfully constructed a novel VLP vaccine targeting the M, F, and HN proteins of the virulent goose-derived NDV NA-1 strain. This vaccine has exhibited the ability to elicit strong humoral and cellular immune responses, consequently providing effective protection against virulent NDV challenges in chickens [13]. However, its immunoprotective effect on waterfowl had not been explored, which was one of the aims of the present study. The VP3 protein is a suitable antigen candidate for the development of novel vaccines or diagnostic methods for GPV due to its high abundance of the three structural proteins of GPV and excellent immunogenicity [21,22]. VLPs assembled from the VP3 protein can induce neutralizing antibodies better than inactivated and attenuated vaccines [24]. Additionally, multivalent vaccines are more advantageous in terms of cost reduction and labor relief. Hence, based on the BES and ND VLPs vector platform, we developed novel ND-GP cVLPs displaying the NDV HN protein and the GPV VP3 protein. The BES and ND VLPs have been extensively utilized in the development of multivalent vaccines. The versatility of NDV VLPs in carrying diverse exogenous proteins, including the AIV HA protein [16], IBD VP2 protein [1], *Brucella* BCSP31 protein [18], and RSV F and G protein [14,15], underscores the repeatability and reproducibility of the ND-GP cVLPs developed in this study. Notably, ND-GP cVLPs are composed of the structural protein M, the highly immunogenic proteins F and VP3, and a lack viral infectious nucleic acid. Compared to attenuated vaccines, ND-GP cVLPs do not pose the risk of viral genomic contamination, which could lead to the evolution of novel viral strains and the reappearance of virulence, thereby offering an eco-friendly farming environment free from viral nucleic acid contamination.

Baculoviruses remaining in the ND-GP cVLPs were not inactivated in this study because they do not infect vertebrates due to their narrow host range and reliance on specific species of invertebrates to provide them with natural nutrients [25]. Additionally, baculovirus activates mouse dendritic cells and induces nonspecific NK cell and T-cell immune responses [26], suggesting that baculovirus can be used as a nonspecific immune promoter. If potential biosafety concerns are considered, baculovirus can be inactivated using formaldehyde, β-propiolactone, diethylenimine, and Triton X-100 [27,28]. Immunization with 40 µg of chimeric ND VLPs containing AIV HA protein has protected chickens from challenge with virulent NDV and H9N2 AIV [16]. Hence, to determine the appropriate immunization dose, we administered three doses of 30, 50, and 70 µg of ND-GP cVLPs. The results indicated that 50 µg of the ND-GP cVLPs induced HI or neutralizing antibody titers compared to the commercial vaccines, while 70 µg of the ND-GP cVLPs produced better antibody titers among all the groups. However, these immunization doses represent preliminary explorations in laboratory studies. The immune dose and immune adjuvant should be optimized to reduce costs, minimize side effects, and increase efficacy in the further studies or clinical trials of ND-GP cVLPs. Cellular immunity was not measured due to the lack of commercial antibodies against goose-derived T-cell surface markers such as CD3, CD4, and CD8. However, the ability of VLPs to induce cellular immune responses has been fully confirmed. VLPs interact with antigen-presenting cells, such as dendritic cells (DCs), stimulating Th1 and Th2 cell immune responses [29,30,31]. Furthermore, in our previous study, we found that ND VLPs are easily recognized and processed by DCs, which induces DC maturation via the TLR4/NF-κB pathway, promotes DC migration via the CCR7-CCL19/CCL21 pathway, and ultimately presents antigens to T cells [32]. Additionally, chimeric ND VLPs containing HA protein have been reported to induce greater T-cell immune responses in the CD3+ CD4+ cells (Th) and CD3+ CD8+ cells (Tc) subsets than commercial vaccines in chickens [16].

The protective effect against challenge is the final indicator used to evaluate the performance of a vaccine. Both the ND-GP cVLPs and the commercial vaccine effectively protected the geese from mortality following the virulent NDV or GPV challenge and alleviated damage caused by the viral infection in target organs. Although the mortality rate among the geese in the PBS group was only 40% after the GPV challenge, which potentially indicated an increased resistance with an advancing goose age, some geese exhibited typical subacute clinical symptoms such as depression, reduced feeding, and lethargy. Notably, the ND-GP cVLPs group outperformed the commercial vaccine group in terms of weight gain post-challenge. This advantage may be attributed to the higher antibody titers generated after the immunization with 70 µg of the ND-GP cVLPs compared to those in the commercial vaccine group, resulting in the improved potency of virus neutralization. Furthermore, the induction of latent cellular immunity by ND-GP cVLPs may also contribute to their superior protective efficacy. Additionally, the positive rate of virus detection in the oropharyngeal and cloacal swabs, as well as the duration of the virus shedding, were lower in the ND-GP cVLPs and commercial vaccine groups than in the PBS group post-challenge. Generally, a stronger humoral and cellular immune response is correlated with reduced viral replication [33]. To summarize, these results indicate that ND-GP cVLPs provide effective protection against virulent NDV and GPV.

## 5. Conclusions

We developed chimeric ND-GP bivalent cVLPs (ND-GP cVLPs) displaying the NDV HN and GPV VP3 proteins. ND-GP cVLP immunization in goslings produced excellent HI antibodies against NDV and neutralizing antibodies against GPV. Additionally, the ND-GP cVLPs provided thorough protection and reduced damage due to the viral infection. Compared to that in the PBS group, the viral shedding in the oropharyngeal and cloacal swabs was decreased after the virulent NDV or GPV challenge. To summarize, this study provides eco-friendly and efficient novel ND-GP cVLPs for preventing goose-derived NDV and GP. The use of ND VLPs as a vector platform for the development of multi-conjugate vaccines has further increased.

## Figures and Tables

**Table 1:** The detection of NDV in the oropharyngeal and cloacal swabs after the challenge.

NDV Challenge	Oropharyngeal Swab					Cloacal Swab				
	3 dpc	5 dpc	7 dpc	9 dpc	11 dpc	3 dpc	5 dpc	7 dpc	9 dpc	11 dpc
ND-GP cVLPs	4/10	4/10	2/10	0/10	0/10	4/10	4/10	1/10	0/10	0/10
Commercial vaccine	6/10	5/10	2/10	1/10	0/10	5/10	5/10	1/10	1/10	0/10
PBS	10/10	NA	NA	NA	NA	9/10	NA	NA	NA	NA

NA represents all the deaths of the experimental birds.

**Table 2:** Detection of GPV in oropharyngeal and cloacal swabs after challenge.

GPV Challenge	Oropharyngeal Swab					Cloacal Swab				
	3 dpc	5 dpc	7 dpc	9 dpc	11 dpc	3 dpc	5 dpc	7 dpc	9 dpc	11 dpc
ND-GP cVLPs	5/10	5/10	3/10	0/10	0/10	6/10	4/10	2/10	0/10	0/10
Commercial vaccine	6/10	5/10	3/10	0/10	0/10	7/10	6/10	3/10	1/10	0/10
PBS	10/10	10/10	8/10	7/10	5/10	10/10	10/10	7/10	7/10	6/10

NA represents all the deaths of the experimental birds.

## Data Availability

The data generated during this study are available at “figshare” at http://doi.org/10.6084/m9.figshare.26488639.

## Supplementary Materials

The following supporting information can be downloaded at: https://www.mdpi.com/article/10.3390/microorganisms12112266/s1. Figure S1: Immunoelectron microscopy observation of ND-GP cVLPs.

## References

[1] Li J., Ding J., Chen K., Xu X., Shao Y., Zhang D., Yu X., Guo C., Qian J., Ding Z. (2024). Protective effects of a novel chimeric virus-like particle vaccine against virulent NDV and IBDV challenge. Vaccine.

[2] Miller P.J., Koch G., Swayne D. (2013). Newcastle Disease. Diseases of Poultry.

[3] Liu X.F., Wan H.Q., Ni X.X., Wu Y.T., Liu W.B. (2003). Pathotypical and genotypical characterization of strains of Newcastle disease virus isolated from outbreaks in chicken and goose flocks in some regions of China during 1985–2001. Arch. Virol..

[4] Xu M., Chang S., Ding Z., Gao H.W., Wan J.Y., Liu W.S., Liu L.N., Gao Y., Xu J. (2008). Genomic analysis of Newcastle disease virus strain NA-1 isolated from geese in China. Arch. Virol..

[5] Wu W., Liu H., Zhang T., Han Z., Jiang Y., Xu Q., Shao Y., Li H., Kong X., Chen H. (2015). Molecular and antigenic characteristics of Newcastle disease virus isolates from domestic ducks in China. Infect. Genet. Evol..

[6] Zhang S., Wang X., Zhao C., Liu D., Hu Y., Zhao J., Zhang G. (2011). Phylogenetic and pathotypical analysis of two virulent Newcastle disease viruses isolated from domestic ducks in China. PLoS ONE.

[7] Xiang B., Chen R., Liang J., Chen L., Lin Q., Sun M., Kang Y., Ding C., Liao M., Xu C. (2020). Phylogeny, pathogenicity and transmissibility of a genotype XII Newcastle disease virus in chicken and goose. Transbound. Emerg. Dis..

[8] Derzsy D. (1967). A viral disease of goslings. I. Epidemiological, clinical, pathological and aetiological studies. Acta Vet. Acad. Sci. Hung..

[9] Gough D., Ceeraz V., Cox B., Palya V., Mato T. (2005). Isolation and identification of goose parvovirus in the UK. Vet. Rec..

[10] Mohsen M.O., Bachmann M.F. (2022). Virus-like particle vaccinology, from bench to bedside. Cell. Mol. Immunol..

[11] Hong Q., Liu J., Wei Y., Wei X. (2023). Application of Baculovirus Expression Vector System (BEVS) in Vaccine Development. Vaccines.

[12] McGinnes L.W., Pantua H., Laliberte J.P., Gravel K.A., Jain S., Morrison T.G. (2010). Assembly and biological and immunological properties of Newcastle disease virus-like particles. J. Virol..

[13] Xu X., Ding Z., Yuan Q., Ding J., Li J., Wang W., Cong Y., Ouyang W., Wang Y., Qian J. (2019). A genotype VII Newcastle disease virus-like particles confer full protection with reduced virus load and decreased virus shedding. Vaccine.

[14] McGinnes Cullen L., Schmidt M.R., Kenward S.A., Woodland R.T., Morrison T.G. (2015). Murine immune responses to virus-like particle-associated pre- and postfusion forms of the respiratory syncytial virus F protein. J. Virol..

[15] Murawski M.R., McGinnes L.W., Finberg R.W., Kurt-Jones E.A., Massare M.J., Smith G., Heaton P.M., Fraire A.E., Morrison T.G. (2010). Newcastle disease virus-like particles containing respiratory syncytial virus G protein induced protection in BALB/c mice, with no evidence of immunopathology. J. Virol..

[16] Xu X., Qian J., Qin L., Li J., Xue C., Ding J., Wang W., Ding W., Yin R., Jin N. (2020). Chimeric Newcastle Disease Virus-like Particles Containing DC-Binding Peptide-Fused Haemagglutinin Protect Chickens from Virulent Newcastle Disease Virus and H9N2 Avian Influenza Virus Challenge. Virol. Sin..

[17] Ogembo J.G., Muraswki M.R., McGinnes L.W., Parcharidou A., Sutiwisesak R., Tison T., Avendano J., Agnani D., Finberg R.W., Morrison T.G. (2015). A chimeric EBV gp350/220-based VLP replicates the virion B-cell attachment mechanism and elicits long-lasting neutralizing antibodies in mice. J. Transl. Med..

[18] Xu X., Ding Z., Li J., Liang J., Bu Z., Ding J., Yang Y., Lang X., Wang X., Yin R. (2019). Newcastle disease virus-like particles containing the Brucella BCSP31 protein induce dendritic cell activation and protect mice against virulent Brucella challenge. Vet. Microbiol..

[19] Liu J., Ren Z., Wang H., Zhao Y., Wilker P.R., Yu Z., Sun W., Wang T., Feng N., Li Y. (2018). Influenza virus-like particles composed of conserved influenza proteins and GPI-anchored CCL28/GM-CSF fusion proteins enhance protective immunity against homologous and heterologous viruses. Int. Immunopharmacol..

[20] Park B.R., Bommireddy R., Chung D.H., Kim K.H., Subbiah J., Jung Y.J., Bhatnagar N., Pack C.D., Ramachandiran S., Reddy S.J.C. (2023). Hemagglutinin virus-like particles incorporated with membrane-bound cytokine adjuvants provide protection against homologous and heterologous influenza virus challenge in aged mice. Immun. Ageing.

[21] Wang J., Cong Y., Yin R., Feng N., Yang S., Xia X., Xiao Y., Wang W., Liu X., Hu S. (2015). Generation and evaluation of a recombinant genotype VII Newcastle disease virus expressing VP3 protein of Goose parvovirus as a bivalent vaccine in goslings. Virus Res..

[22] Yin X., Zhang S., Gao Y., Li J., Tan S., Liu H., Wu X., Chen Y., Liu M., Zhang Y. (2012). Characterization of monoclonal antibodies against waterfowl parvoviruses VP3 protein. Virol. J..

[23] OIE (2012). Newcastle disease. Manual of Diagnostic Tests and Vaccines for Terrestrial Animals: Mammals, Birds and Bees.

[24] Ju H., Wei N., Wang Q., Wang C., Jing Z., Guo L., Liu D., Gao M., Ma B., Wang J. (2011). Goose parvovirus structural proteins expressed by recombinant baculoviruses self-assemble into virus-like particles with strong immunogenicity in goose. Biochem. Biophys. Res. Commun..

[25] Targovnik A.M., Simonin J.A., Mc Callum G.J., Smith I., Cuccovia Warlet F.U., Nugnes M.V., Miranda M.V., Belaich M.N. (2021). Solutions against emerging infectious and noninfectious human diseases through the application of baculovirus technologies. Appl. Microbiol. Biotechnol..

[26] Suzuki T., Chang M.O., Kitajima M., Takaku H. (2010). Baculovirus activates murine dendritic cells and induces non-specific NK cell and T cell immune responses. Cell. Immunol..

[27] Rueda P., Fominaya J., Langeveld J.P., Bruschke C., Vela C., Casal J.I. (2000). Effect of different baculovirus inactivation procedures on the integrity and immunogenicity of porcine parvovirus-like particles. Vaccine.

[28] Duan K., Tang X., Zhao J., Ren G., Shao Y., Lu T., He B., Xu L. (2022). An inactivated vaccine against infectious pancreatic necrosis virus in rainbow trout (*Oncorhynchus mykiss*). Fish Shellfish Immunol..

[29] Zhao C., Ao Z., Yao X. (2016). Current Advances in Virus-Like Particles as a Vaccination Approach against HIV Infection. Vaccines.

[30] Al-Barwani F., Donaldson B., Pelham S.J., Young S.L., Ward V.K. (2014). Antigen delivery by virus-like particles for immunotherapeutic vaccination. Ther. Deliv..

[31] Moffat J.M., Cheong W.S., Villadangos J.A., Mintern J.D., Netter H.J. (2013). Hepatitis B virus-like particles access major histocompatibility class I and II antigen presentation pathways in primary dendritic cells. Vaccine.

[32] Qian J., Xu X., Ding J., Yin R., Sun Y., Xue C., Wang J., Ding C., Yu S., Liu X. (2017). Newcastle disease virus-like particles induce DC maturation through TLR4/NF-kappaB pathway and facilitate DC migration by CCR7-CCL19/CCL21 axis. Vet. Microbiol..

[33] Yang M., Lai H., Sun H., Chen Q. (2017). Virus-like particles that display Zika virus envelope protein domain III induce potent neutralizing immune responses in mice. Sci. Rep..

