# Supplementary material for: Development of a Novel Chimeric ND-GP cVLPs Vaccine for the Prevention of Goose-Derived Newcastle Disease and Gosling Plague
Source: Microorganisms. 2024 Nov 8;12(11):2266. doi: 10.3390/microorganisms12112266 (PMC11596917; doi:10.3390/microorganisms12112266)
Supplement: Supplementary file 1 [file microorganisms-12-02266-s001.zip › microorganisms-3257635-supplementary.pdf]

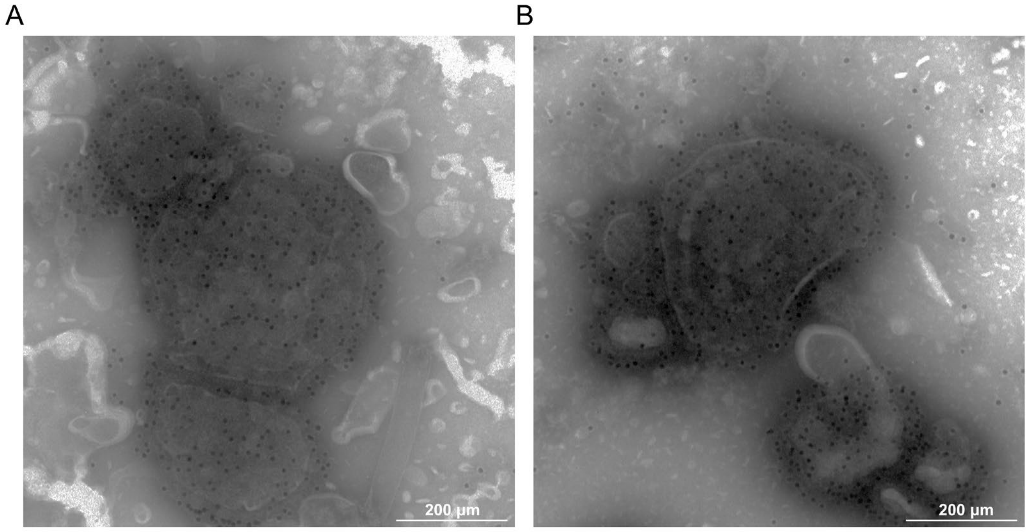

Supplement Fig.1. Immunoelectron microscopy observation of ND-GP cVLPs. Observe the 10 nm gold-labeled NDV HN protein (A) and GPV VP3 protein (B) on the surface of ND-GP cVLPs.
